# Efficient route for preparation of Nd^3+^ doped Y_2_O_3_ nanoparticles at intermediate temperature

**DOI:** 10.1016/j.heliyon.2024.e25864

**Published:** 2024-02-06

**Authors:** M.M. Arghavan, A.A. Sabouri-Dodaran, M. Sasani Ghamsari

**Affiliations:** a Department of Physics, Payame Noor University, P.O.Box 19395-3697, Tehran, Iran; b Photonics and Quantum Technologies Research School, Nuclear Science and Technology Research Institute, 11155-3486, Tehran, Iran

**Keywords:** Yttrium oxide, Neodymium ions, Middle temperature, Nanoparticles

## Abstract

Yttrium oxide nanoparticles are one of the proper host materials for rare-earth elements. The rare-earth ions doped in yttrium oxide nanoparticles have medical applications such as biological imaging and photonic applications such as waveguides in the infrared region, laser mediums, sensitizers, LEDs, etc. The preparation of rare-earth ion-doped Y_2_O_3_ nanoparticles is usually done through the solid-state process and at a very high temperature, such as 1200 °C. In this research, using the solid-state process and during multi-step heat treatment at temperatures of 210 °C, 380 °C, and 500 °C, white nanopowders of Nd:Y_2_O_3_were prepared in 6 h. The produced nanopowders were studied using various characterization methods. The results showed that the produced nanopowders have a cubic structure and an average particle size between 22 and 65 nm.

## 1 Introduction

**Fig. 1:**
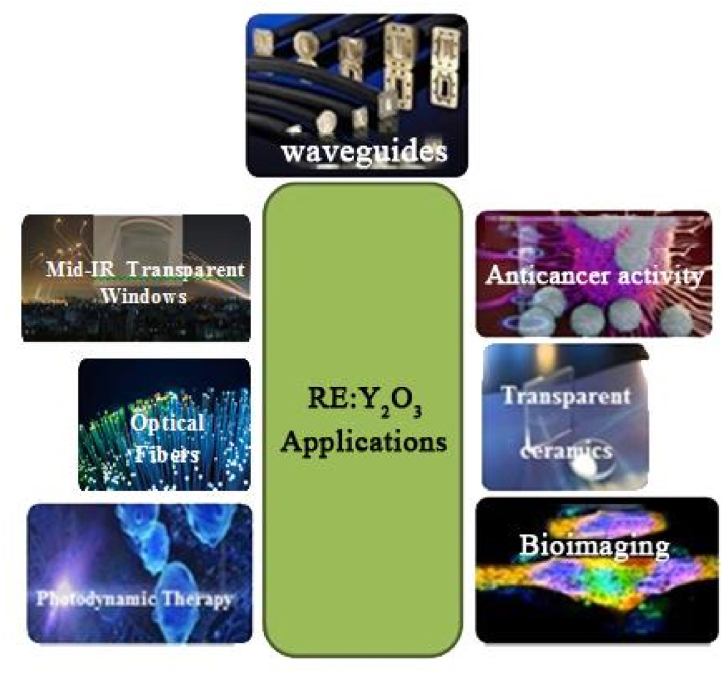
Different rare earth doped yttrium oxide applications.

Yttrium oxide nanoparticles, Y_2_O_3_, due to their thermal stability, stable chemical-mechanical properties, high dielectric constant, low phonon energy and suitable ionic and crystal structure, have been used as one of the best host environments for rare earth (RE) ions such as Tb^3+^, Eu^3+^, La^3+^,etc. The RE-doped yttrium oxide nanoparticles have a strong luminescence effect and have many medical applications, including biological imaging, photodynamic therapy, etc. Also, due to their surface-to-volume ratio, rare earth ions such as Nd^3+^-doped yttrium oxide nanoparticles can be used in photonic applications such as optical fibers, waveguides in the infrared region, optical amplifiers, solid-state laser mediums, mechanical applications, electrostatics, synthesis of inorganic compounds, etc. [[1], [2], [3], [4], [5]]. In Fig. 1, some of its applications were shown.

Yttrium oxide, with or without RE-dopants, is synthesized by different methods such as hydrothermal, microwave irradiation, precipitation, combustion, sol-gel, etc., which leads to different structures and morphologies such as thin films, nanoneedles, nanocompoits, etc. Recently, Rao et al. synthesized yttrium oxide nanoneedles by the CO_2_ carbonization method. Also, Liu et al. improved the quality of yttrium oxide thin films by CVD. Yttria Compounds with RE-dopants, such as lanthanum-doped yttrium oxide nanoceramics, have also been synthesized by the scientists [[6], [7], [8], [9], [10], [11], [12], [13], [14], [15]]. RE-doped yttria nanopowders have been synthesized by several methods, such as hydrolysis, combustion, high-temperature solid-state reactions, sol-gel, precipitation, molten salt, solvothermal, hydrothermal, etc. In almost all the mentioned methods of producing yttrium oxide with or without RE-dopants, due to the amorphous nature of as-prepared samples, high-temperature crystallization reaches up to 1700 °C to obtain a highly crystalline state, which directly leads to an increase in the average particle size [[1], [2], [3],[16], [17], [18], [19], [20], [21], [22], [23], [24], [25], [26], [27], [28], [29], [30], [31], [32], [33]]. Of course, Chen et al. reported the production of yttria in micron dimensions at a temperature of 600 °C [20]. Among the methods mentioned, the solid-state process, which is a common method to produce RE-doped yttria nanopowders, has been developed recently [34,35]. By this method, it is possible to prepare nanoparticles with a clear surface, controlled shape, high productivity, and prevent particle agglomeration. Also, this process is simple, has high yield, high selectivity, and less pollution. It must be noted that the solid-state process is very slow, needs a lot of energy, and occurs at high temperatures [[36], [37], [38], [39], [40]]. In this article, for the first time, an optimized solid-state process for Nd^3+^-doped yttrium oxide nanoparticles was developed at a much lower temperature and in less time than other reported procedures, and then their optical and structural properties were characterized by various methods such as X-ray diffraction, Fourier transform infrared (FTIR), field emission scanning electron microscope (FE-SEM), transmission electron microscopy (TEM), Raman spectroscopy, X-ray photoelectron spectroscopy (XPS), Energy-dispersive X-ray analysis (EDAX) and Selected Area Electron Diffraction (SAED) pattern.

## 2 Experimental procedure

To prepare Nd^+3^:Y_2_O_3_ nanopowders, 0.6 mmol of yttrium nitrate hexahydrate (Y(NO_3_)_3_·6H_2_O,99.8%, Sigma Aldrich) and 0.05 mmol of neodymium nitrate hexahydrate (Nd(NO_3_)_3_·6H_2_O,99.8%, Sigma Aldrich) were added to a stirring environment that includes deionized water and ethanol alcohol (99.9%) with 1:1 ratio. Then, the oxalic acid (C_2_H_2_O_4_,98%, Sigma-Aldrich), and glycine (C_2_H_5_NO_2_, ≥ 99%, Sigma-Aldrich) with 2:1 ratio were added to the ingredients. The produced solution was stirred for a few minutes, and after that, it was poured into a crucible. Then, it was placed in the oven at 210 °C for about 2 h. After that, the resulting black-brown powder was placed in the 380 °C oven for about 2 h. Finally, the resulting brown powder was placed in the 500 °C oven for 2 h. Now, the resulting white powders are Nd^3+^:Y_2_O_3_, in which their optical properties will be characterized as follows. First, in order to identify the experimental accuracy and characterize the Nd^3+^:Y_2_O_3_ nanocrystal, an X-ray diffraction pattern was applied with a PANalytical (XPertPRo MPD) diffractometer. TEM images, which are produced by TEM Philips EM 208S-100kv device, are used to collect the structural, chemical, and morphological information of the nanoparticles. Also, energy-dispersive X-ray analysis (EDAX) is used for the elemental analysis or chemical characterization of the sample. The field emission scanning electron microscope (FE-SEM) method is applied using a FE-SEM ZEISS Sigma 300 device to examine the substructures of the sample. The Fourier transform infrared (FTIR) spectrum is plotted using a Bruker Alpha II FTIR spectrometer which determines the chemical bond fluctuations and examines the structure and purity of the sample. Also, a Raman Takram P50C0R10 spectrometer is applied to characterize the structural and phase transitions of the sample. Finally, to determine the elemental species of the sample, the XPS pattern as a surface chemical analysis technique, plotted by BESTEC (EA 10) device.

## 3 Result and discussion

**Fig. 2:**
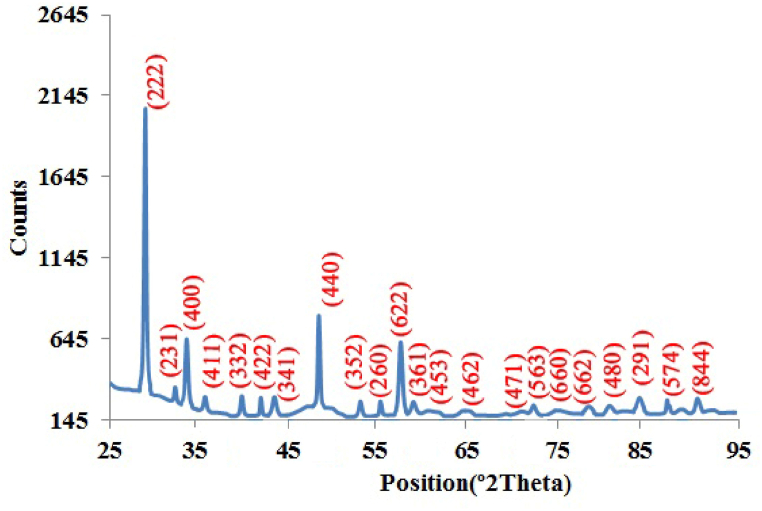
X-ray diffraction pattern of Nd:Y_2_O_3_nanopowders. The peak with the maximum intensity is located at 29.2°.

Fig. 2 shows the X-ray diffraction pattern of the sample with the Miller indices of the sample using Fullprof software. It also shows the agreement with the International Center for Diffraction Data (ICDD) data set (ref. code: 01-073-1334), which is related to the cubic structure of Nd^+3^:Y_2_O_3_. The crystal parameters of the sample are a = 10.604 Å, b = 10.604 Å, c = 10.604 Å, α = 90°, β = 90°, and γ = 90°. The significant peaks are located at angles of 29.2°, 33.6°, 48.3°, and 57.4° respectively.

**Fig. 3:**
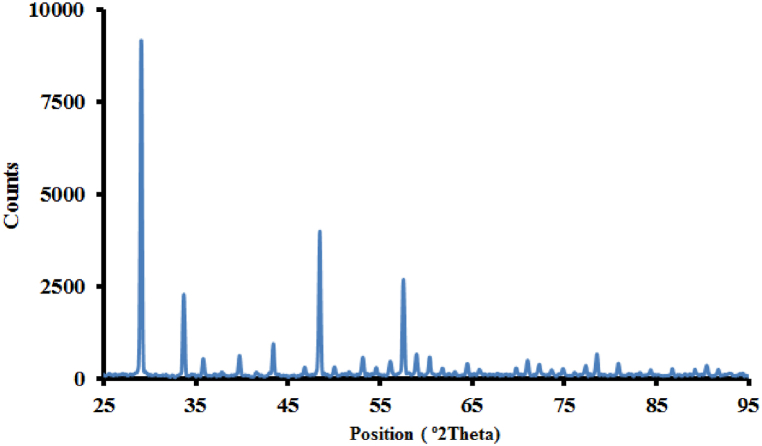
X-ray diffraction pattern of synthetized sample of this report, which annealed at 1200 °C.

Also, by using the XRD pattern and Scherrer's equation (Eq. 1), the dimension of the crystal particles can be determined as follows [41]:

(1) D=(k×λ)/(FWHM×Cosθ)

where D is the size of the crystal, k is the shape constant, which is approximately equal to 0.89, λ is the incident wavelength, which in this article is the wavelength of copper, and is equal to 1.5406 Å. θ and FWHM are the incident angle and the full width at half the maximum of the diffraction peak, respectively. According to Scherer's equation, the size of the crystallites is about 33.5 nm. Fig. 3 shows the XRD pattern of the synthesized Nd:Y_2_O_3_ nanopowders of this report, which annealed at 1200 °C in 60 min. By using Scherrer's equation, the average size of the particles is 55 nm which indicates that the particle size increases with increasing temperature.

**Fig. 4:**
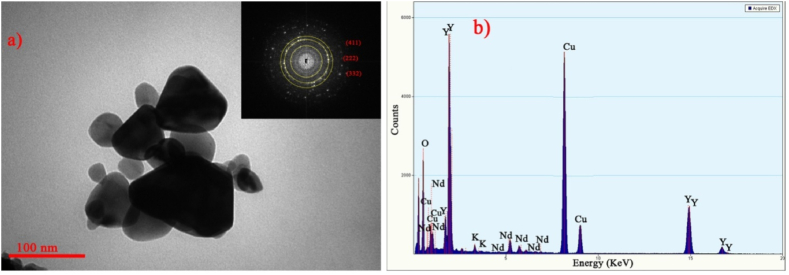
a) TEM image and SAED pattern of Nd:Y_2_O_3_ nanoparticles. The dimensions of the particles are approximately 25 nm. b) EDAX analysis of the sample shows the presence of Nd^3+^ ions.

The small difference between the intensity and position of some peaks in Fig. 2, Fig. 3 is due to the XRD imaging of different crystallographic plates in the two samples and also the increase in the size of the particles caused by the increase in the annealing temperature to 1200 °C. TEM pattern in Fig. 4(a) indicates the shape and size of neodymium-doped yttrium oxide nanopowders. Also, EDAX analysis in Fig. 4(b) shows the presence of Nd^3+^ions. The Cu and K peaks are related to the analysis device. It can be seen that the peaks of the XRD pattern of the sample and indexed spots ((222), (411) and (332)) in the Selected Area Electron Diffraction (SAED) pattern which is shown in Fig. 4(a), match well with each other. According to the TEM images of the sample, the average size of the particle is about 47 nm, which is consistent with the X-ray diffraction spectrum and Scherer's calculations.

**Fig. 5:**
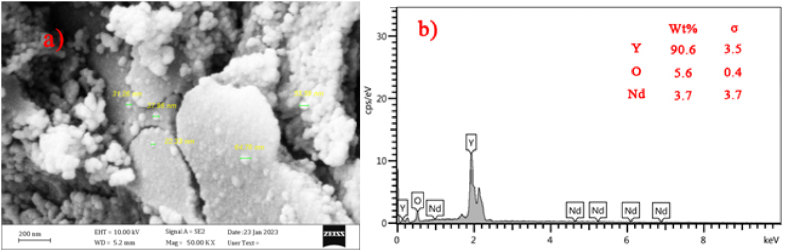
a) FE-SEM image of Nd:Y_2_O_3_ nanoparticles. The dimensions of the particles are between 22 and 65 nm. b) EDAX analysis of the sample that shows the presence of Nd^3+^ ions.

Fig. 5 shows the FE-SEM image and EDAX analysis of Nd:Y_2_O_3_ nanoparticles. It shows that the size of the particles is between 22 and 65 nm (Fig. 5(a)). The presence of the Nd^+3^ ions in the structure of the sample can be identified according to EDAX analysis (Fig. 5(b)).

**Fig. 6:**
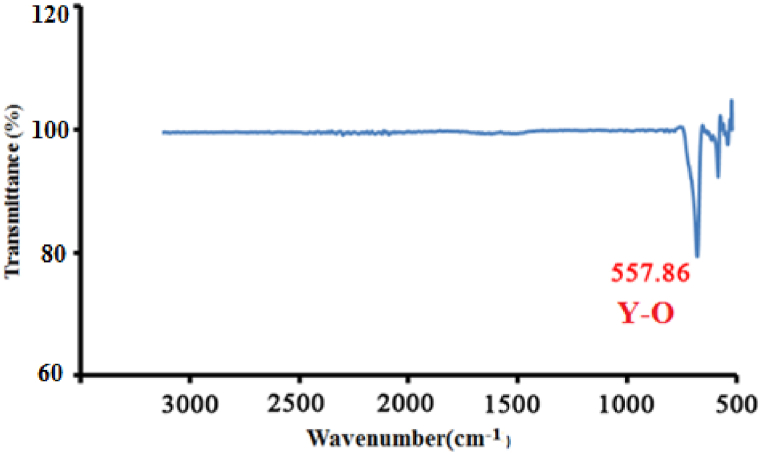
FTIR curve of neodymium doped yttrium oxide nanoparticles. The Y–O bond is shown in a wavenumber of 557.86 cm^−1^.

Fig. 6 shows the FTIR curve of the Nd:Y_2_O_3_ nanopowders that was plotted using the oscillation, approximately located at 558 cm^−1^, indicates the presence of Y–O bonds in the sample.

**Fig. 7:**
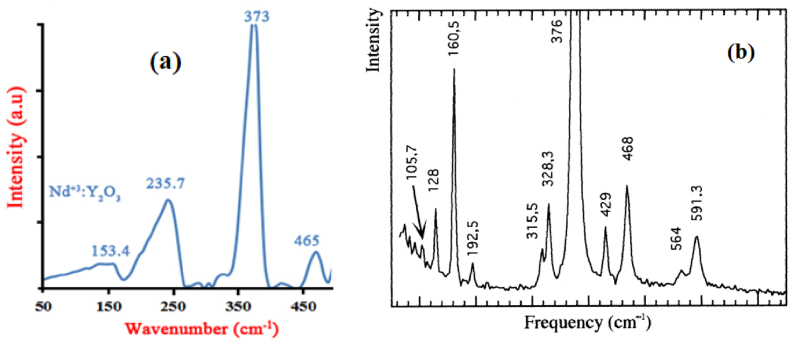
a) Raman spectra of neodymium-doped yttrium oxide nanoparticles and b) yttrium oxide that reproduced from Ref. [44].

Fig. 7 shows the Raman spectra of Y_2_O_3_ and Nd:Y_2_O_3_ nanoparticles which is excited by 532 nm. The Raman spectrum peaks of yttrium oxide, which are located at 129 and 318, have the E_g_ mode, and the peaks located at 161, 193, 329, 376, 429, 469, 564, and 591 cm^−1^have the E_g_ + A_g_ mode. Furthermore, Fig. 6 shows the Raman spectrum peaks of Nd:Y_2_O_3_, which are located at 153.4, 235.7, 373, 456, 513.6, 555.9, and 597.9 cm^−1^. In particular, the peaks at 153.4, 235.7, and 465 cm^−1^ are attributed to Nd–O bonds, and 373 cm^−1^ is related to Y–O bonds. The difference and similarity between the location and intensity of Raman spectrum peaks of pure Y_2_O_3_ and Nd:Y_2_O_3_ confirm the successful doping process of Nd^3+^ and the cubic symmetry and Ia-3 space group of Nd:Y_2_O_3_, respectively [[42], [43], [44], [45]].

**Fig. 8:**
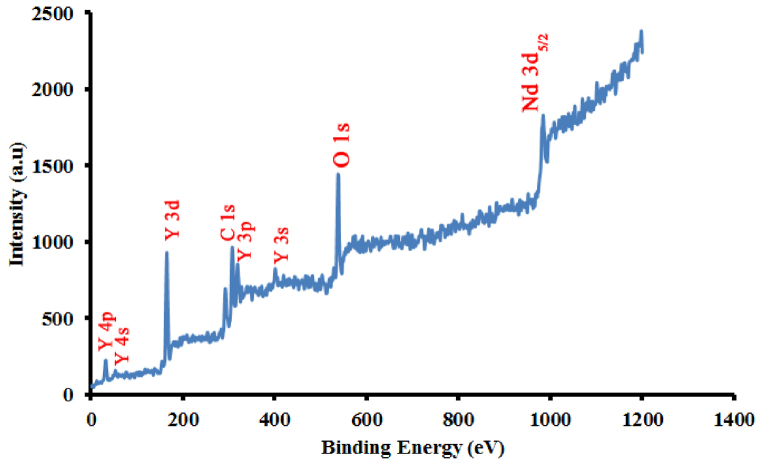
XPS spectra of Nd^+3^:Y_2_O_3_ nanopowders.

In the XPS spectra (Fig. 8), the peaks at 32.13, 63.53, 165.22, 307.28, 319.24, 401.48, 539.06, and 984.68 eV correspond to Y4p, Y4s, Y3d, C1s, Y3p, Y3s, O1s, and Nd 3d_5/2_ respectively. It should be noted that the binding energy of the electron is also dependent on the quantum numbers.

Therefore, in Fig. 8, next to the symbol of each element, these numbers are also marked. For example, next to the Nd element symbol, the principal quantum number, orbital quantum number, and spin quantum number corresponding to the specified bonding energy are equal to 3, 2, and 1/2, respectively.

## 4 Conclusion

In this report, we synthesized the neodymium-doped yttrium oxide nanoparticles during three temperature steps (up to 500 °C) by using the optimized middle temperature solid-state process to produce the proper crystallinity and reduce the cost and energy. In the following, the sample was characterized to show the successfully performed process. The XRD pattern of the sample with Miller indices confirms the structure of Nd:Y_2_O_3_ nanopowders. TEM and FE-SEM are also applied to show the dimension and morphology of the nanocrystals. Also, it was shown that their results matched Scherrer's equation. It was shown that SAED and XRD patterns match as well. The FTIR curve of the sample obviously shows the Y–O bands and ensures that the synthesis has been done properly. Also, XPS, as a well-known surface-sensitive characterization method, with Raman spectrum, have obviously shown the presence of Y,O, and Nd elements in the sample.

## Data availability statement

-Sharing research data helps other researchers evaluate your findings, build on your work and to increase trust in your article. We encourage all our authors to make as much of their data publicly available as reasonably possible. Please note that your response to the following questions regarding the public data availability and the reasons for potentially not making data available will be available alongside your article upon publication.

Has data associated with your study been deposited into a publicly available repository?

-No, data was used for the research described in the article.

## CRediT authorship contribution statement

**M.M. Arghavan:** Writing – original draft, Methodology, Investigation, Formal analysis, Data curation, Conceptualization. **A.A. Sabouri-Dodaran:** Writing – review & editing, Visualization, Supervision, Project administration. **M. Sasani Ghamsari:** Writing – review & editing, Validation, Supervision, Resources, Methodology, Funding acquisition.

## Declaration of competing interest

We wish to confirm that there are no known conflicts of interest associated with this manuscript and there has been no significant financial support for this work that could have influenced its outcome.

## References

[1] Lamnini S., Elsayed H., Lakhdar Y., Baino F., Smeacetto F., Bernardo E. (2022). Robocasting of advanced ceramics: ink optimization and protocol to predict the printing parameters-A review. Heliyon.

[2] Lin L., Starostin S.A., Li S., Khan S.A., Hessel V. (2017). Synthesis of yttrium oxide nanoparticles via a facile microplasma-assisted process. Chem. Eng. Sci..

[3] Loitongbam R.S., Singh W.R., Phaomei G., Singh N. Sh (2013). Blue and green emission from Ce^3+^ and Tb^3+^ co-doped Y_2_O_3_ nanoparticles. J. Lumin..

[4] Mao Y., Huang J.Y., Ostroumov R., Wang K.L., Chan J.P. (2008). Synthesis and luminescence properties of erbium-doped Y_2_O_3_ nanotubes. J. Phys. Chem. C..

[5] Ukare R.S., Kurzekar R.R., Zade G.D., Dhoble S.J. (2018). Yttrium oxide as an engineering material. Int. J. Curr. Eng. Sci. Res..

[6] Mishra M., Kuppusami P., Sairam T.N., Singh A., Mohandas E. (2011). Effect of substrate temperature and oxygen partial pressure on microstructure and optical properties of pulsed laser deposited yttrium oxide thin films. Appl. Surf. Sci..

[7] Govindasamy R., Mao L., Bao T., Wen W., Wang Sh, Gomathi Th, Gnanasundaram N., Rebezov M., Shariati M.A., Chung I.-M., Thiruvengadam M., Zhang X. (Mar. 2021). Yttrium oxide nanoparticle synthesis: an overview of methods of preparation and biomedical applications. Appl. Sci..

[8] Wu C.-H., Chen J.-Z. (2015). Ultrafast atmospheric-pressure-plasma-jet processed conductive plasma-resistant Y_2_O_3_/carbon-nanotube nanocomposite. J. Alloys Compd..

[9] Nunes D., Pimentel A., Matias M., Freire T., Araújo A., Silva F., Gaspar P., Garcia S., Carvalho P.A., Fortunato E., Martins R. (2019). Tailoring upconversion and morphology of Yb/Eu doped Y_2_O_3_ nanostructures by acid composition mediation. Nanomater.

[10] Huang Y., Jiang D., Zhang J., Lin Q. (2009). Fabrication of lanthanum doped yttria transparent ceramics. Chin. Sci. Bull..

[11] Devaraju M.K., Yin Sh, Sato T. (2009). A fast and template free synthesis of Tb:Y_2_O_3_ hollow microspheres via supercritical solvothermal method. Cryst. Growth Des..

[12] Rao M., Lai A., Zan M., Gao M., Xiao Y. (2022). Synthesis of yttrium oxide nanoneedles with carbon dioxide carbonization. Nanomater.

[13] Liu L., Kawaharamura T., Sakamoto M., Nishi M., Dang G.T., Sato Sh (2021). The quality improvement of yttrium oxide thin films grown at low temperature via the third-generation mist chemical vapor deposition using oxygen-supporting sources. P.S.S.b.

[14] Mao Y., Engels J., Houben A., Rasinski M., Steffens J., Terra A., Linsmeier Ch, Coenen J.W. (2017). The influence of annealing on yttrium oxide thin film deposited by reactive magnetron sputtering: process and microstructure. Nucl. Mater. Energy.

[15] Pearce S.J., Parker G.J., Charlton M.D.B., Wilkinson J.S. (2010). Structural and optical properties of Yttrium Oxide thin films for planar waveguiding applications. J. Vac. Sci..

[16] Mariscal-Becerra L., Flores-Jimenez M.C., Alcantara J.M.H., Camarillo E., Falcony-Guajardo C., Vazquez-Arreguin R., Sanchez H.M. (2018). Structural and luminescent analysis of hafnium-doped yttrium oxide and yttrium-doped hafnium oxide powders and doped with trivalent europium and terbium ions. J. Nanophotonics.

[17] Nagajyothi P.C., Pandurangan M., Veerappan M., Kim D.H., Sreekanth T.V.M., Shim J. (2018). Green synthesis, characterization and anticancer activity of yttrium oxide nanoparticles. Mater. Lett..

[18] Aghazadeh M., Ghaemi M., Nozad Golikand A., Yousefi T., Jangju E. (2011). Yttrium oxide nanoparticles prepared by heat treatment of cathodically grown yttrium hydroxide. ISRN ceramics.

[19] Mellado-Vázquez R., García-Hernández M., López-Marure A., Yolanda López-Camacho P., de Jesús Morales-Ramírez A., Isaac Beltrán-Conde H. (2014). Sol-gel synthesis and antioxidant properties of yttrium oxide nanocrystallites incorporating P-123. Materials.

[20] Chen K., Peng J., Srinivasakannan C., Yin Sh, Guo Sh, Zhang L. (2018). Effect of temperature on the preparation of yttrium oxide in microwave field. J. Alloys Compd..

[21] Huang Z., Sun X., Xiu Zh, Chen Sh, Tsai Ch-T. (Jun. 2004). Precipitation synthesis and sintering of yttria nanopowders. Mater. Lett..

[22] Kumar J.B.P., Ramgopal G., Vidya Y.S., Anantharaju K.S., Daruka Prasad B., Sharma S.C. (2015). Green synthesis of Y_2_O_3_:Dy nanophosphor with enhanced photocatalytic activity. SAA: Molecular and Biomolecular Spectroscopy.

[23] Zhai Y., Yao Z., Ding S., Qiu M., Zhai Z. (2003). Synthesis and characterization of Y_2_O_3_:Eu nanopowder via EDTA complexing sol–gel process. Mater. Lett..

[24] Chiechio R.M., Battaglia R., Caponnett A., Butera E., Franzò G., Reitano R. (2023). Er:Y_2_O_3_ and Nd:Y_2_O_3_ nanoparticles: synthesis, pegylation, characterization and study of their luminescence properties. Chemosensors.

[25] Kodaira C.A., Stefani R., Maia A.S., Felinto M.C.F.C., Brito H.F. (2007). Optical investigation of Y_2_O_3_: Sm^3+^ nanophosphor prepared by combustion and Pechini methods. J. Lumin..

[26] Mangalaraja R.V., Mouzon J., Hedstrom P., Kero I., Ramam K.V.S., Camurri Carlos P. (2008). Combustion synthesis of Y_2_O_3_ and Yb–Y_2_O_3_ Part I. Nanopowders and their characterization. J. Mater. Process. Technol..

[27] Diego-Rucabado A., Candela M.T., Aguado F., González J., Rodríguez F., Valiente R. (2020). A comparative study on luminescence properties of Y_2_O_3_: Pr^+3^ nanocrystals prepared by different synthesis methods. Nanomaterials.

[28] Venkatachalam N., Saito Y., Sogaz K. (2009). Synthesis of Er^3+^ doped Y_2_O_3_ nanophosphors. J. Am. Ceram. Soc..

[29] Singh V., Dabre K.V., Dhoble S.J., Lakshminarayana G. (2020). Green emitting holmium (Ho) doped yttrium oxide (Y_2_O_3_) phosphor for solid state lighting. OptikOptik (Stuttg.).

[30] Rodriguez S.V., Villabona-Leal E.G., Mixteco-Sánchez J.C., Romero V.H., Desirena H., Perez E. (2018). Study of visible light emission under UV excitation in Y_2_O_3_:Er^3+^-Gd^3+^ and Y_2_O_3_:Eu^3+^-Gd^3+^ nanocrystals. JSST.

[31] Tamrakar R.K., Dubey V. (2016). Synthesis, structural characterization and thermoluminescence glow curve study of gadolinium-doped Y_2_O_3_nanophosphor. JTUSCI.

[32] Mishra K., Singh S.K., Singh A.K., Rai S.B. (2013). Frequency upconversion in Er^3+^ doped Y_2_O_3_nanophosphor: Yb^3+^ sensitization and tailoring effect of Li^+^ion. Mater. Res. Bull..

[33] Lukyashin K.E., Chepusov A.S., Solomonov V.I. (2020).

[34] Zhang M., Wang Z., Zuo H. (2013). Interactions between Y_2_O_3_–Al mixture studied by solid-state reaction method. Vacuum.

[35] Kruk A., Ziewiec K. (2022). Preparation, characterization and magneto-optical properties of Sm-doped Y_2_O_3_ polycrystalline material. Micromachines.

[36] Solgi S., Sasani Ghamsari M., Tafreshi M.J., Karvane R. (2020). Synthesis condition effects on the emission enhancement of YBO_3_ powder. Optik.

[37] Solgi S., Tafreshi M.J., Sasani Ghamsari M. (2019). A facile route for synthesis of highly pure α-CaB_4_O_7_ compound. Mater. Res. Express..

[38] Solgi S., Tafreshi M.J., Sasani Ghamsari M. (2019). A novel method for synthesis of CaB_4_O_7_ compound using ammonium pentaborate. Mater. Res. Express..

[39] Solgi S., Sasani Ghamsari M. (2023). Facile route for synthesis of strontium hexaborate crystalline powders. Int. J. Appl. Ceram. Technol..

[40] Dao-hua L., Shao-fen H., Jie Ch, Cheng-yan J., Cheng Y. (2017). Solid-state chemical reaction synthesis and characterization of lanthanum tartrate nanocrystallites under ultrasonication spectra. MSEB.

[41] Muchuweni E., Sathiaraj T.S., Nyakotyo H. (2017). The Synthesis and characterization of zinc oxide thin films for optoelectronic applications. Heliyon.

[42] Gadipelly Th, Dasgupta A., Sornadurai D., Dhara S. (2021). Controlling and morphology of nanocrystalline Y(OH)_3_ powders synthesized by microwave-hydrothermal route and effect of annealing. Appl. Phys. A..

[43] Sure J., Mishra M., Tarini M., Shankar A.R., Krishna N.G., Kuppusami P., Mallik C., Mudali U.K. (2013). Microstructural characterization and chemical compatibility of pulsed laser deposited yttria coatings on high density graphite. Thin Solid Films.

[44] Husson E., Proust C., Gillet P., Itié J.P. (Sep.–Oct. 1999). Phase transitions in yttrium oxide at high pressure studied by Raman spectroscopy. Mater. Res. Bull..

[45] Umesh B., Eraiah B., Nagabhushana H., Sharma S.C., Nagabhushana B.M., Shivakumara C., Rao J.L., Chakradhar R.P.S. (2012). Structural, EPR, Optical and Raman studies of Nd_2_O_3_:Cu^2+^nanophosphors. Spectrochim. Acta - A: Mol. Biomol. Spectrosc.

